# 17*β*-Estradiol Regulates Microglia Activation and Polarization in the Hippocampus Following Global Cerebral Ischemia

**DOI:** 10.1155/2018/4248526

**Published:** 2018-04-18

**Authors:** Roshni Thakkar, Ruimin Wang, Jing Wang, Ratna K. Vadlamudi, Darrell W. Brann

**Affiliations:** ^1^Department of Neuroscience and Regenerative Medicine, Medical College of Georgia, Augusta University, Augusta, GA, USA; ^2^Department of Neurobiology, North China University of Science and Technology, Tangshan, Hebei, China; ^3^Department of Obstetrics and Gynecology, University of Texas Health, San Antonio, TX, USA

## Abstract

17*β*-Estradiol (E2) is a well-known neuroprotective hormone, but its role in regulation of neuroinflammation is less understood. Recently, our lab demonstrated that E2 could regulate the NLRP3 (NOD-like receptor protein 3) inflammasome pathway in the hippocampus following global cerebral ischemia (GCI). Here, we examined the ability of E2 to regulate activation and polarization of microglia phenotype in the hippocampus after global cerebral ischemia (GCI). Our *in vivo* study in young adult ovariectomized rats showed that exogenous low-dose E2 profoundly suppressed microglia activation and quantitatively shifted microglia from their “activated,” amoeboid morphology to a “resting,” ramified morphology after GCI. Further studies using M1 “proinflammatory” and M2 “anti-inflammatory” phenotype markers showed that E2 robustly suppressed the “proinflammatory” M1 phenotype, while enhancing the “anti-inflammatory” M2 microglia phenotype in the hippocampus after GCI. These effects of E2 may be mediated *directly* upon microglia, as E2 suppressed the M1 while enhancing the M2 microglia phenotype in LPS- (lipopolysaccharide-) activated BV2 microglia cells *in vitro*. E2 also correspondingly suppressed proinflammatory while enhancing anti-inflammatory cytokine gene expression in the LPS-treated BV2 microglia cells. Finally, E2 treatment abolished the LPS-induced neurotoxic effects of BV2 microglia cells upon hippocampal HT-22 neurons. Collectively, our study findings suggest a novel E2-mediated neuroprotective effect via regulation of microglia activation and promotion of the M2 “anti-inflammatory” phenotype in the brain.

## 1. Introduction

The steroid hormone, 17*β*-estradiol (E2), is known to have multiple actions on various tissues of the body, including the brain. A neuroprotective effect for E2 was first suggested from studies in female gerbils, which found that females had lower incidence and less damage following ischemic injury as compared to males [1]. Likewise, studies using animal models of stoke and traumatic brain injury (TBI) found similar sex differences in brain injury, with young adult females displaying less neuronal damage and greater survival rates compared to young adult males [2–5]. Since then, various studies have confirmed neuroprotective effects of E2 in both focal cerebral ischemia and global cerebral ischemia (CGI) models [6–8]. Subsequent studies done in humans have also documented that women are more protected against stroke than men, at least until the age of menopause, when the circulating estrogen levels fall [9]. Furthermore, a neuroprotective role for E2 has also been suggested in other neurodegenerative disorders such as Parkinson's disease, Alzheimer's disease, and multiple sclerosis [6, 10–18].

Several mechanisms have been implicated to mediate E2 neuroprotection in the brain. For instance, our group and others have shown that E2 neuroprotection can involve genomic signaling, nongenomic signaling, antioxidative functions, and regulation of mitochondrial bioenergetics, as well as anti-inflammatory actions [19–24]. The classical estrogen receptors, ER-*α* and ER-*β*, as well as the new putative G-protein coupled estrogen receptor 1 (GPER1), have been implicated to mediate E2 neuroprotection in the brain [6, 14, 18, 25–34]. Numerous studies also implicated a role for E2 neuroprotective actions in the brain by upregulation of prosurvival factors and downregulation of proapoptosis factors and attenuation of NADPH activity and oxidative stress, as well as reduction of glutamate toxicity [12, 25, 35–38]. However, paradoxically comparatively less is known about the anti-inflammatory role of E2 in the brain, which could also contribute to the neuroprotective actions of E2.

Inflammation in the central nervous system involves responses from resident immune cells, microglia, inflammasomes, and downstream inflammatory cytokine production [39, 40]. Furthermore, microglia cells polarize into either an M1 (proinflammatory) phenotype, or an M2 (more anti-inflammatory, repair-like) phenotype [41, 42]. The polarization status of these cells can be induced by certain factors and can be characterized by the type of M1- or M2-specific markers expressed. For instance, “M1” microglia phenotype can be induced *in vitro* by lipopolysaccharide (LPS) or interferon-*γ* (IFN*γ*) and express destructive proinflammatory factors/markers such as tumor necrosis factor alpha (TNF-*α*), interleukin 1 beta (IL-1*β*), and inducible nitric oxide synthase (iNOS) [43–45]. In contrast, interleukin-4 (IL-4) and IL-10 have been shown to induce the alternative “M2” phenotype that possesses neuroprotective/anti-inflammatory/repair properties and can be characterized by expression of key markers such as arginase-1, CD206, chitinase 3-like 3 (Ym1), and interleukin 1 receptor antagonist (IL1RA) [45]. Alterations in microglial phenotype have been suggested to play a role in multiple neurological disorders such as focal stroke, Alzheimer's disease (AD), and multiple sclerosis [46–48].

Recent studies from our lab have shown that E2 can exert anti-inflammatory effects to suppress activation of the nod-like receptor protein 3 (NLRP3) inflammasome pathway in the hippocampus following GCI in ovariectomized rats [23]. We further showed that E2 reduces NLRP3 pathway molecules, including NLRP3, apoptosis-associated speck-like protein containing a CARD (caspase recruitment domain) [49], cleaved caspase 1, and IL-1*β* [23]. Other studies in rodents have also confirmed E2 inhibition of inflammasome and proinflammatory cytokines in models of focal ischemic injury, spinal cord injury, depression, and amyotrophic lateral sclerosis [24, 50–54]. In addition to inflammasome regulation, E2 has also been shown to regulate microglia activation after central nervous system (CNS) injury and in various neurodegenerative disorders [55–58]. Furthermore, sex and age differences in microglia activation in mice occur after focal ischemic injury, where young adult females had less microglia activation as compared to young males [59]. A more recent study also reported that E2, via GPER1, can regulate microglia activation and proinflammatory cytokine production after GCI [60]. Finally, *in vitro* studies indicate that E2 can regulate microglia phagocytic activity and inhibit production of proinflammatory TNF-*α* and IL-1*β* after hypoxia and can upregulate anti-inflammatory TREM2 (triggering receptor expressed on myeloid cells 2) and IL-10 [61, 62]. While our understanding of the anti-inflammatory effects of E2 is increasing, the field still lacks a clear understanding of whether E2 can regulate microglia polarization and dynamics in the hippocampus *in vivo* following GCI. To address this deficit in our knowledge, we performed a detailed *in vivo* analysis of M1, proinflammatory, and M2, anti-inflammatory, microglia phenotype markers in the hippocampus following GCI and determined the regulatory effect of E2. We also examined the changes in morphology of microglia after GCI, with and without E2 replacement, as this has been shown to correlate with activation status of microglia. Furthermore, we conducted in vitro studies utilizing the BV2 microglia cell line to more easily examine potential *direct* anti-inflammatory effects of E2 upon microglia. BV2 cells are an immortalized murine microglial cell line frequently used to study microglial function and potential direct effects of factors upon microglia [43, 63]. To activate BV2 microglia cells, we chose LPS, the most widely used activator and inducer of M1 microglial phenotype and inflammatory actions in microglia [43]. Stimulation of microglia cells with LPS is often used to mimic aspects of CNS inflammation as it causes a rapid increase of expression and release of proinflammatory mediators. Furthermore, the response of BV2 microglia cells to LPS activation has also been shown to be highly similar to activation of primary microglia, as evidenced by gene and protein expression profiling, as well as functional capacity for inflammation (e.g., cytokine expression, M1 phenotype induction, and cell to cell interaction). Thus, use of BV2 cells and LPS provided a very reproducible and robust model for induction of M1 phenotype and an inflammatory activation profile of BV2 microglia cells. This *in vitro* model therefore allowed us to determine whether E2 could act *directly* upon microglia cells to regulate microglial polarization, pro- and anti-inflammatory cytokine gene expression, and the neurotoxic ability of activated microglia.

## 2. Methods

### 2.1. Animals and Surgical Procedures

**Figure 1:**
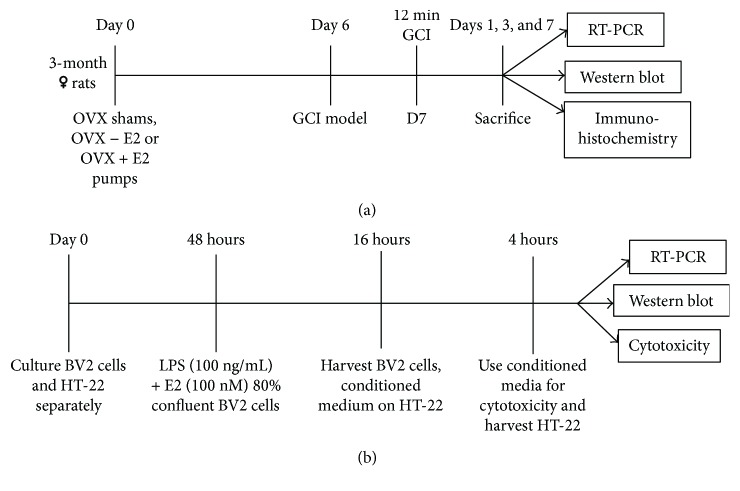
Experimental design used for *in vivo* and *in vitro* studies. (a) Young adult female rats were ovariectomized (OVX) at day 0, and the E2 group was administered with E2 pumps. The four-vessel occlusion GCI model followed this at days 6 and 7. The animals were then sacrificed at the desired time points after ischemia, and the samples were processed as needed. (b) The BV2, murine microglial cell line and hippocampal cell line, HT-22 were cultured up to 80% confluence. The BV2 cells were treated with LPS or LPS + E2 for 16 hours and harvested for further analyses. The conditioned media from these cells was taken and used to treat HT-22 cells for four hours. The conditioned media were then taken for LDH assay, and the HT-22 cells were harvested for further analyses.

Augusta University Institutional Animal Care and Use Committee (IACUC) approved all animal procedures. The studies were conducted in accordance with National Institutes of Health guidelines for animal research. Three-month-old young, adult, female, Sprague Dawley rats were housed under normal conditions in the Augusta University's animal housing facility with two rats per cage. There was free access to chow and water for the rats, and lighting conditions were from 7 am to 7 pm. The animals were routinely monitored before and after the surgery. Rats were bilaterally ovariectomized under isoflurane anesthesia and separated into four groups—shams, estrogen (E2), global cerebral ischemia (GCI) injury, and GCI injury with estrogen (E2) treatment groups. The two E2 treatment group animals were immediately administered with 17*β*-estradiol dissolved in 20% *β*-cyclodextrin added to minipumps (0.0167 mg E2 in 20% *β*-cyclodextrin, 0.5 *μ*L/hr, 14-day release; Alzet, Cupertino, CA). The vehicle used was 20% *β*-cyclodextrin. Pumps were placed in the upper mid-back region to allow subcutaneous administration of E2. Previous studies by our group have shown that this dose of E2 generates physiological Diestrus I levels of circulating E2 (10–15 pg/mL) [12]. All rats, except for sham and E2 controls, were subjected to GCI via the 4-vessel occlusion method [64] after 7 days of ovariectomy. One day prior to occlusion, that is, 6 days after ovariectomy, all animals were anesthetized using ketamine/xylazine (10 : 1, 100 mg/mL, 0.1 mL per 100 gm of rat's body weight was injected intraperitoneally (IP)), their vertebral arteries were electrocauterized, and the common carotid arteries were exposed. Twenty-four hours later, the animals were anesthetized using 1–4% isoflurane anesthesia, and then the common carotid arteries were transiently occluded with hemostatic clips for 12 minutes for all animals except the shams. Sham and E2 control animals had their arteries exposed but not occluded. Ischemia-reperfusion was allowed to occur, and animals were checked for loss of their righting reflex within 30 seconds and pupil dilation for successful GCI. Animals were sacrificed using transcardial perfusion and decapitation at 1, 3, and 7 days after GCI (Figure 1(a)). Each group had 7 animals to begin with; there were zero deaths in the sham group, two animals died in the GCI group, and one animal in the GCI + E2 group. All surgeries and experiments were repeated in triplicate.

### 2.2. Tissue Collection

All animals were transcardially perfused and decapitated at the desired time point after GCI. Brains were dissected in the midsagital plane and fixed in 4% paraformaldehyde for 24 hours, cryoprotected in 30% sucrose, and sectioned on a cryostat to obtain 20-micron-thick hippocampal sections. These sections were then used for immunofluorescence staining. For RT-PCR and Western blot analysis, brains were collected and the hippocampal tissue was dissected out, frozen, and processed for either RNA isolation or homogenized for detection of proteins via Western blot analysis.

### 2.3. *In Vitro* Cell Cultures

Figure 1(b) illustrates the experimental design used for the *in vitro* studies utilized in our study. BV2 microglial cells [43] were cultured in sterile RPMI medium with 5% fetal bovine serum and 1% penicillin/streptomycin antibiotic at 37°C in a 5% CO_2_ incubator. After the cells were 80% confluent, they were divided into three groups: control, LPS, and LPS + E2. The control group received no treatment, and cells were allowed to grow in complete medium. The LPS group received LPS treatment (100 ng/mL) [65] for 16 hours, and the LPS + E2 group received LPS plus 100 nM E2 [55, 56] treatment for 16 hours. The cells were then observed at 20x magnification under a bright-field microscope, and images were analyzed for number of active cells depending on their amoeboid/rounded shape per microscopic field. Amoeboid/rounded shape of BV2 cells is indicative of their activated state [66]. The cells were then harvested for RNA or protein isolation. Neurotoxicity studies were done using the HT-22 mouse hippocampal neuronal cell line [67]. HT-22 cells were treated with conditioned media from the control, LPS, and LPS + E2 groups of BV2 cells for 4 hours. Conditioned media were then tested for cytotoxicity using LDH assay, and cell lysates were used to test for apoptosis from conditioned media-treated HT-22 cells. The LDH assay was performed using the Pierce LDH Cytotoxicity Assay Kit (Thermo Scientific, Prod number 88954), as per the manufacturer's protocol.

### 2.4. RT-PCR

Hippocampal tissue samples or BV2 cells were collected, and RNA was isolated using the SV total RNA isolation system (Promega). The RNA was then used for the reverse transcriptase PCR reaction using the Superscript III one-step RT-PCR system with platinum Taq DNA Polymerase (Invitrogen) and respective primers as listed in Table 1 for *in vivo* samples and Table 2 for *in vitro* samples (Integrated DNA Technologies). The gene expression analyses were done using the comparative ΔΔCt method. The mRNA level changes were expressed as a fold change as compared to the sham animals for *in vivo* or the control group for *in vitro*. All Ct values for target genes were normalized to CypA gene for *in vivo* samples [68] and 18S for *in vitro* samples.

### 2.5. Western Blot Analysis

Hippocampal tissue after GCI or BV2 and HT-22 cells' sample were collected as mentioned above. Individual samples were homogenized in RIPA buffer, the homogenates were centrifuged at 13,000 rpm for 10 minutes at 4°C, and the supernatants were used for protein estimation by Lowry's Assay (Lowry's Assay Kit, Sigma). Thirty micrograms of protein for each sample was separated on 12% sodium dodecyl sulfate-polyacrylamide gel electrophoresis, transferred on nitrocellulose membrane, and blocked with 5% bovine serum albumin for 1 hour at room temperature with gentle shaking. Blocking was followed by incubation with primary antibodies, mouse monoclonal CD68 (Abcam, ab31630, 1 : 500), goat polyclonal CD206 (Santa Cruz Biotechnology, sc-34577, 1 : 100), rabbit monoclonal iNOS (Cell signaling, D6B6S, 1 : 1000), rabbit polyclonal Ym1 (StemCell Technologies, 01404, 1 : 800), IL-1*β* (Abcam, ab9722, 1 : 500), and rabbit polyclonal cleaved caspase-3 (Asp175) (Cell Signaling, 9661, 1 : 1000), overnight at 4°C with gentle shaking. Glyceraldehyde 3-phosphate dehydrogenase (GAPDH, Santa Cruz Biotechnology, sc-32233, 1 : 2000) was used as a loading control. The membrane was then washed with TBST buffer to remove unbound primary antibody and incubated with secondary Alexa Fluor 680 or 800 anti-rabbit/goat/mouse IgG (1 : 4000) for 1 hour at room temperature with gentle shaking. Blots were scanned using Odyssey Imaging System (LI-COR Bioscience, Lincoln, NB). The intensity of bands was quantified using ImageJ software. The immunoblot data was corrected for corresponding GAPDH values and presented as fold change in protein as compared to the sham animals or control group.

### 2.6. Immunofluorescence Staining

Twenty *μ*m thick coronal sections were washed with PBS and 0.4% Triton-X PBS for 20 minutes. The sections were then blocked with 10% normal donkey serum for 1 hour at room temperature in PBS containing 0.1% Triton X-100, followed by incubation with primary antibody for 2 nights at 4°C in the same buffer. Primary antibodies used for this study included mouse monoclonal CD68 (Abcam, ab31630, 1 : 500), goat polyclonal CD206 (Santa Cruz Biotechnology, sc-34577, 1 : 50), rabbit monoclonal iNOS (Cell signaling, D6B6S, 1 : 400), rabbit monoclonal IL1RA (Abcam, ab124962, 1 : 400), and goat polyclonal Iba1 (Abcam, ab5076, 1 : 400). After primary antibody incubation, sections were washed for 3 × 10 minutes at room temperature, followed by incubation with the appropriate secondary antibody: Alexa-Fluor488/568/647 donkey anti-rabbit/anti-mouse/anti-goat (1 : 200) (Invitrogen) RT/1 hour. Sections were then washed with PBS containing 0.1% Triton X-100 for 3 × 10 min, followed by 2 × 5 min with 1x PBS and briefly with distilled H_2_O. The sections were then mounted with water-based mounting medium containing antifading agents and observed using confocal microscopy. All images were captured on a confocal laser microscope (Carl Zeiss, Germany) using the Zen software at 40x magnifications and 50 *μ*m scale bar.

### 2.7. Confocal Microscopy Image Analysis

**Figure 2:**
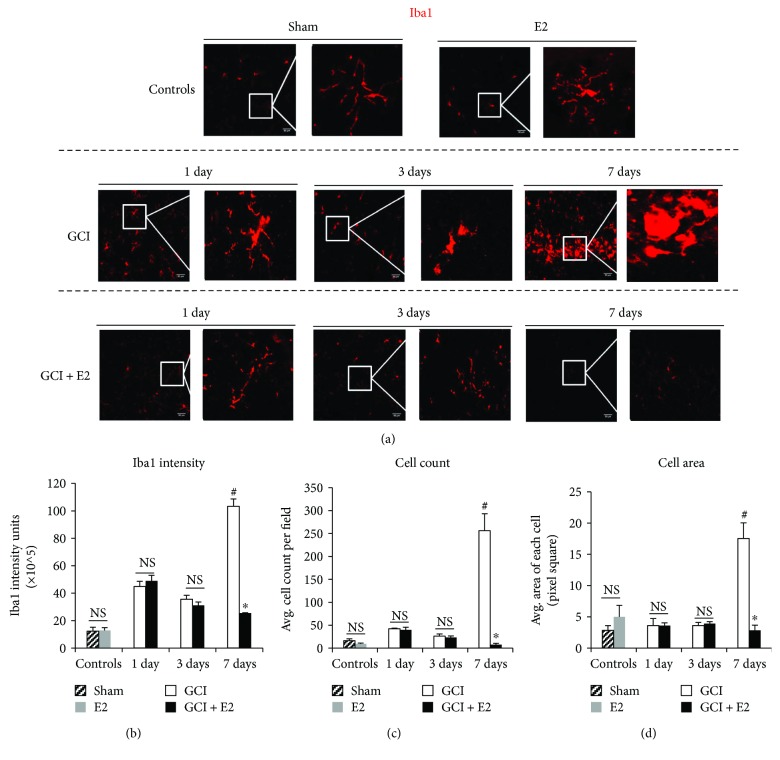
Temporal pattern of microglia activation and morphological changes in the hippocampus after global cerebral ischemia and its regulation by estrogen. (a) Representative confocal images show Iba1 staining of microglia cells in the hippocampal CA1 region of control animals (sham and E2) and at days 1, 3, and 7 after global cerebral ischemia (GCI). This activation and morphological changes are suppressed under the effect of E2 as shown in the lower panel (magnification = 40x, scale bar = 50 *μ*m). (b) Intensity quantification of Iba1 staining of controls and at days 1, 3, and 7 with and without E2 treatment after GCI. (c) Microglia cell count of controls and at days 1, 3, and 7 with and without E2 treatment after GCI. (d) Average cell area of controls and at days 1, 3, and 7 with and without E2 treatment after GCI (*n* = 5‐6 animals per group) (^#^*p* < 0.05, controls, 1 and 3 day GCI versus 7 day GCI, ^∗^*p* < 0.05, 7 day GCI versus 7 day GCI + E2, NS = not significant).

The intensity of all confocal images was quantified using ImageJ software (downloaded from http://www.imagej.nih.gov). Each individual image that was quantified in ImageJ had 1024 × 1024 pixel size and was subjected to a 50.0 pixel background subtraction for all images irrespective of the antibody and/or group they belonged to. This confirmed consistency in analysis as well as allowed for a uniform threshold to be set for intensity quantification. The images were then individually analyzed for quantified amount of intensity via the “Analyze” tool of ImageJ. The results are represented as average intensity of all the images per group. Furthermore, for Iba1-stained rat brain sections (Figure 2), the microglia morphology analyses and cell counting were performed using ImageJ. Cell area in terms of pixel square specifically for quantitative morphology analyses was accepted as morphology quantification criteria as per recent description by Fernández-Arjona et al. [69]. Both counting and cell area were determined by converting the 1024 × 1024 pixel image into 16-bit, then setting the Otsu's method threshold, previously used by our lab for image analyses [23]. Once the threshold was set, the exact area of each counted cell as well as the total cell count per image was obtained using the “analyze particles” tool. The quantification was plotted as average number of cells per field in every group and average area of cells per image in every group. These quantifications further affirm the visual observations of morphology changes as per Kreutzberg's classification [41].

### 2.8. Statistical Analysis

The ANOVA tests, and/or independent two-sample *t*-test, were used for testing the significance where appropriate. Two factors were taken into consideration throughout the study, a group factor (sham or E2 control, GCI and GCI + E2, or control, LPS, and LPS + E2) and time factor (1 day, 3 days, and 7 days). Appropriate ANOVA test was performed for group factor analyses and interactions. Here, the group factor was considered as a nominal (categorical) variable, and the protein/intensity value was considered as a continuous variable. After confirming the *F* values and *p* values (*p* < 0.05) for each ANOVA output, post hoc test such as Bonferroni's test was conducted to make multiple pairwise comparisons between each of the groups. Specific *F* and *p* values have been mentioned throughout the Results below for further details. All tests were conducted at a 5% level of significance (*p* < 0.05) using the IBM SPSS software (version 23). Data are expressed as mean + standard error (SE).

## 3. Results

### 3.1. E2 Suppresses Microglia Activation and Morphology Changes in the Hippocampus after Global Cerebral Ischemia

Microglia, the resident immune cells of the brain, are the first responders to any type of injury in the brain. We therefore examined the spatial and temporal patterns of microglia activation in the hippocampal CA1 region of control animals (sham and E2), injured animals at days 1, 3, and 7 following GCI as well as GCI + E2 treatment (Figure 2). As shown in Figure 2(a), visual observation suggests that cells transform from a more ramified, thinner process, resting stage to a more rounded, amoeboid-like-activated stage following GCI. This observation is made with reference to Kreutzberg's classification [41]. Maximum activation was seen at day 7 after GCI and was not observed in the control groups as well as in the GCI + E2-treated groups. Moreover, the activated microglia were specifically observed in the hippocampal CA1 region only. Next, these visual observations in microglia activation and morphology were quantified using the following criteria: (i) increased intensity of Iba1 staining, as demonstrated by quantification of confocal images in Figure 2(b) (ANOVA *F* value = 96.281, *p* = 0.000), (ii) average microglia cell count per microscopic field for each group (Figure 2(c)) (ANOVA *F* value = 18.909, *p* = 0.000), and (iii) average area of cells (pixel square) for each group (Figure 2(d)) (ANOVA *F* value = 17.020, *p* = 0.000). Using these criteria, we found maximum activation of microglia cells in the hippocampal CA1 region at day 7 after GCI reperfusion. Furthermore, E2 treatment led to significant suppression of microglia activation as determined by decreased Iba1 expression, reduced cell count, and a change in morphology like reduced amoeboid-like-activated microglia in the GCI + E2 group, as compared to the GCI group at 7 days after GCI (*p* = 0.000) (Figures 2(b)–2(d)). This pattern is consistent with our previous findings, where we showed maximum activation of a key inflammatory pathway, NLRP3 inflammasomes, at day 7 after GCI, and this enhanced activation was significantly suppressed by E2 [23]. No significant suppression of microglia by E2 was reported at days 1 and 3 after GCI (*p* = 1.000) (Figures 2(b)–2(d)). Moreover, it should also be noted that as shown in Figures 2(b)–2(d), E2 control treatment had no effects on microglia polarization states. E2 control treatment without GCI showed no significant changes in Iba1 expression, cell count, or cell area, as compared to sham controls. These results demonstrate that E2 does not affect basal microglia activation, but profoundly suppresses microglia activation in the hippocampus at 7 days after GCI.

### 3.2. E2 Suppresses M1 Microglia Polarization while Enhancing M2 Microglia Polarization in the Hippocampus after GCI

**Figure 3:**
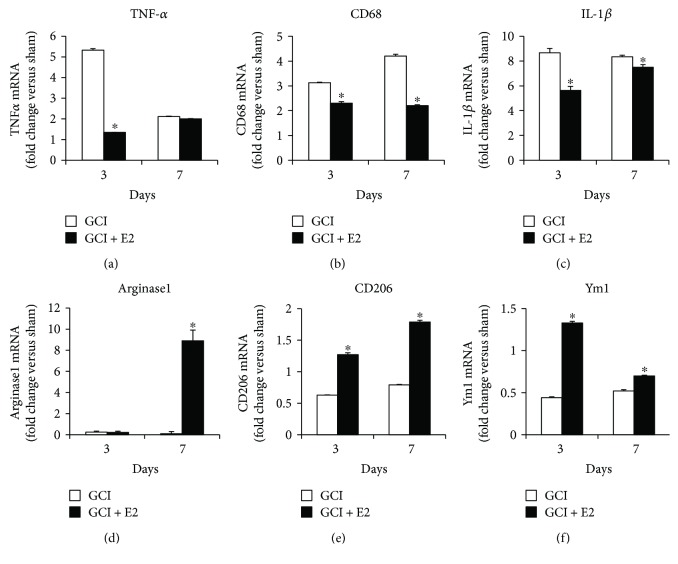
Estrogen suppresses gene expression of M1 markers and upregulates gene expression of M2 markers in the hippocampus after global cerebral ischemia. (a–c) mRNA samples from the hippocampus at days 3 and 7 were collected and analyzed for gene expression of M1, proinflammatory markers, TNF-*α*, CD68, and IL-1*β*. E2 treatment significantly suppressed gene expression of these markers. (d–f) mRNA samples from the hippocampus at days 3 and 7 were collected and analyzed for gene expression of M2, anti-inflammatory markers, Arginase1, CD206, and Ym1. E2 treatment significantly upregulated gene expression of these markers (*n* = 5-6 animals per group) (^∗^*p* < 0.05, GCI versus GCI + E2).

Since microglia activation and morphological changes were suppressed by E2 treatment after GCI, we hypothesized that E2 treatment may result in changes in M1 and M2 microglia polarization in the hippocampus after GCI. Microglia cells are broadly classified into two polarization states: an M1, proinflammatory phenotype, and an M2, anti-inflammatory phenotype. This classification depends upon the expression of specific M1 or M2 markers at a defined time point after exposure to insult. We therefore next examined the gene expression of M1 markers, TNF-*α*, CD68, and IL-1*β*. As shown in Figures 3(a)–3(c), gene expression of these markers showed a 2- to 8-fold increase at days 3 and 7 after GCI, as compared to the sham control group. As also shown in Figures 3(a)–3(c), the increase in gene expression of these M1 markers, TNF-*α* (3 days, *p* < 0.001), CD68 (3 days *p* = 0.001, 7 days *p* < 0.001), and IL-1*β* (3 days *p* < 0.001, 7 days *p* = 0.004), is significantly suppressed by E2 treatment. We next examined gene expression changes for the M2 markers, Arginase1, CD206, and Ym1, after GCI. As shown in Figures 3(d)–3(f), gene expression for the M2 markers shows no increase in expression at days 3 and 7 after GCI, as compared to the sham control group. However, E2 treatment caused a robust increase in gene expression of M2 markers, Arginase1 (7 days *p* < 0.001), CD206 (3 days *p* < 0.001, 7 days *p* < 0.001), and Ym1 (3 days *p* < 0.001, 7 days *p* < 0.001) anti-inflammatory phenotype markers, as compared to the GCI group.

**Figure 4:**
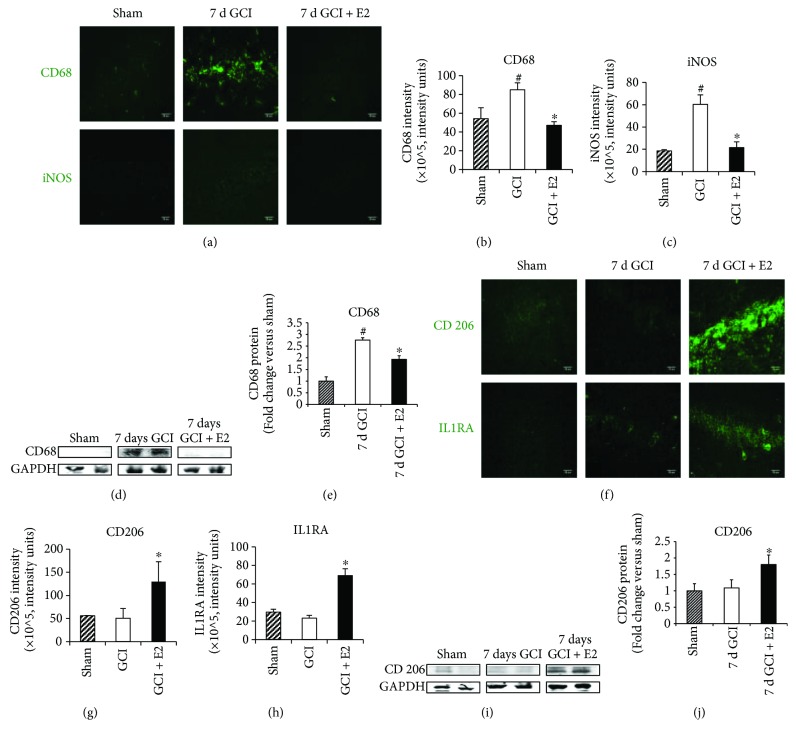
Estrogen suppresses protein levels of M1 markers and upregulates protein levels of M2 markers in the hippocampus after global cerebral ischemia. (a) Representative confocal microscopy images of M1 markers, CD68 and iNOS, in shams and GCI as well as GCI + E2 treatment groups at 7 days after GCI indicate upregulation after GCI and suppression under the effect of E2 (magnification = 40x, scale bar = 50 *μ*m). (b, c) Quantification of intensity of confocal microscopy staining in 5A (*n* = 5-6 animals per group). (d, e) Western blot analysis of classical M1 marker, CD68, in sham and GCI as well as GCI + E2 treatment groups at 7 days after GCI. Quantification of blots indicates a significant increase in CD68 after GCI and suppression under the effect of E2. (f) Representative confocal microscopy images of M2 markers, CD206 and IL1RA, in shams and GCI as well as GCI + E2 treatment groups at 7 days after GCI indicate downregulation after GCI and upregulation under the effect of E2 (magnification = 40x, scale bar = 50 *μ*m). (g, h) Quantification of intensity of confocal microscopy staining in 5F. (i, j) Western blot analysis of classical M2 marker, CD206, in sham, GCI, and GCI + E2 treatment groups at 7 days after GCI. Quantification of Western blots shows that there is not a change in CD206 expression after GCI. However, E2 treatment with GCI leads to a significant upregulation of CD206 (*n* = 5-6 animals per group) (^#^*p* < 0.05, sham versus GCI, ^∗^*p* < 0.05, GCI versus GCI + E2).

To further confirm these changes in M1/M2 polarization after GCI and E2 treatment, we next examined changes in the protein expression of the M1 and M2 markers in the hippocampus after GCI using immunohistochemistry and Western blot analysis. As shown in Figure 4(a), confocal microscopy of immunofluorescence staining of M1 markers, CD68 and iNOS, indicated an increased expression of the M1 markers in the hippocampal CA1 region at day 7 after injury, as compared to the sham control group. Interestingly, this increase in immunostaining for CD68 and iNOS was suppressed by E2 treatment. Quantification of the immunohistochemistry results is shown in Figures 4(b) and 4(c) (ANOVA: 4B *F* value = 11.457, *p* = 0.004, 4C *F* value = 13.310, *p* = 0.002), which confirmed a significant increase in CD68 (*p* = 0.019) and iNOS (*p* = 0.002) immunostaining intensity levels after GCI and a significant decrease of CD68 (*p* = 0.009) and iNOS (*p* = 0.025) by E2 treatment. We next used Western blot analysis to confirm the change in CD68, which showed the highest increase after GCI. As shown in Figure 4(d) and (4e) (ANOVA: 4E *F* value = 38.430, *p* = 0.000), Western blot analysis confirmed a robust increase of CD68 protein levels in the hippocampus at 7 days after GCI, as compared to the sham control group (*p* = 0.000). Furthermore, E2 treatment strongly attenuated the elevation of CD68 protein levels after GCI (*p* = 0.018).

Figure 4(f) shows immunohistochemical examination of the M2 markers, CD206 and IL1RA, in the hippocampus at 7 days after GCI and the effect of E2 treatment. As shown in Figure 4(f), confocal microscopy of immunofluorescence staining for CD206 and IL1RA indicated that immunostaining levels for these markers were strongly elevated by E2 treatment, as compared to the GCI group. Quantification of the M2 marker immunohistochemistry results is shown in Figures 4(g) and 4(h) (ANOVA: 4G *F* value = 9.811, *p* = 0.029, 4H *F* value = 23.759, *p* = 0.000), which confirmed a significant increase in CD206 (*p* = 0.03) and IL1RA (*p* = 0.000) immunostaining intensity levels after E2 treatment. We next used Western blot analysis to confirm the E2 elevation of CD206, which was the highest changed M2 marker protein by immunostaining. As shown in Figures 4(i) and 4(j) (ANOVA: 4J *F* value = 11.025, *p* = 0.004), Western blot analysis further confirmed that E2 induced a robust increase of CD206 (*p* = 0.012) protein levels in the hippocampus after GCI. These results indicate that E2 can suppress the M1, proinflammatory microglia phenotype, and enhance the M2, anti-inflammatory, repair microglia phenotype, in the hippocampus after GCI.

### 3.3. E2 Directly Regulates M1/M2 Microglial Polarization and Cytokine Expression in Activated BV2 Microglia Cells *In Vitro*

**Figure 5:**
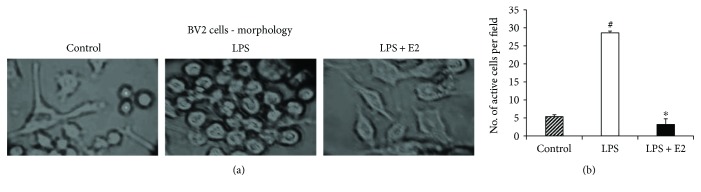
LPS-activated BV2 microglia cells show morphological differences in terms of activation under the effect of E2 *in vitro*. (a) Representative bright field microscopy images show the morphological differences between nonactivated control, LPS-activated and activated + E2-treated BV2 microglia cells *in vitro*. The control group showed longer processes and less rounded cells. The LPS-activated group showed more rounded cells with no thinner processes. The LPS + E2-treated groups further suppress the BV2 cell activation. (b) Quantitative measurement of BV2 cell activation in terms of number of active cells per field (*n* = 5-6 per group) (^#^*p* < 0.05, control versus LPS, ^∗^*p* < 0.05, LPS versus LPS + E2).

To enhance our understanding of whether E2 can act directly on microglia to regulate M1/M2 polarization, we performed *in vitro* experiments using a murine microglial cell line, BV2 cells [43]. BV2 cells were activated using 100 ng/mL lipopolysaccharide (LPS) treatment for 16 hours overnight. The LPS + E2 treatment group was treated with 100 nM E2 in addition to LPS. Morphological examination of control, LPS-activated, and LPS + E2-treated BV2 cells is depicted in representative photomicrographs in Figure 5(a). As shown in Figure 5(a), the LPS-activated cells were round and had an absence of thin processes, indicating an “active stage” phenotype. In contrast, the control and E2-treated LPS-activated BV2 cells showed less rounded and more elongated cells with thinner processes (as compared to LPS-only-treated cells), which is indicative of “resting stage” microglia. A quantitative assessment of total number of activated cells in each group (Figure 5(b)) (ANOVA: *F* value = 177.394, *p* = 0.000) indicates that LPS-activated BV2 cells showed maximum number of amoeboid cells per field. LPS + E2 treatment led to a significant suppression of activated cell count (*p* < 0.001). Based upon these morphological assessments [61], the findings suggest that E2 can act directly upon BV2 microglia cells to regulate their activation.

**Figure 6:**
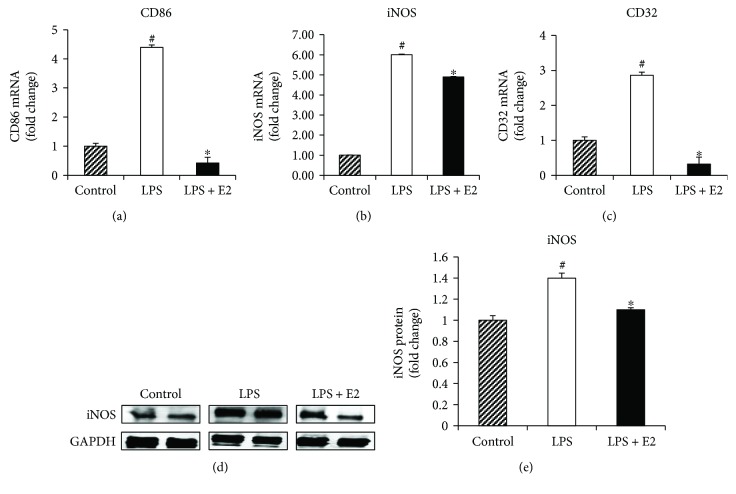
Suppression of M1 phenotype markers by E2 in the LPS-activated BV2 microglia cells *in vitro*. (a–c) mRNA was collected from control, LPS-activated, and LPS activated + E2-treated BV2 cells at 16 hours after activation and treatment. Quantitative RT-PCR analysis of M1 markers, CD86, iNOS, and CD32, indicates a significant upregulation after LPS activation. This upregulation is significantly suppressed by E2 treatment in the activated cells. (d, e) Western blot analysis and quantification of the M1 marker, iNOS, indicate a significant increase in expression after LPS activation and suppression by E2 treatment (*n* = 5-6 per group) (^#^*p* < 0.05, control versus LPS, ^∗^*p* < 0.05, LPS versus LPS + E2).

**Figure 7:**
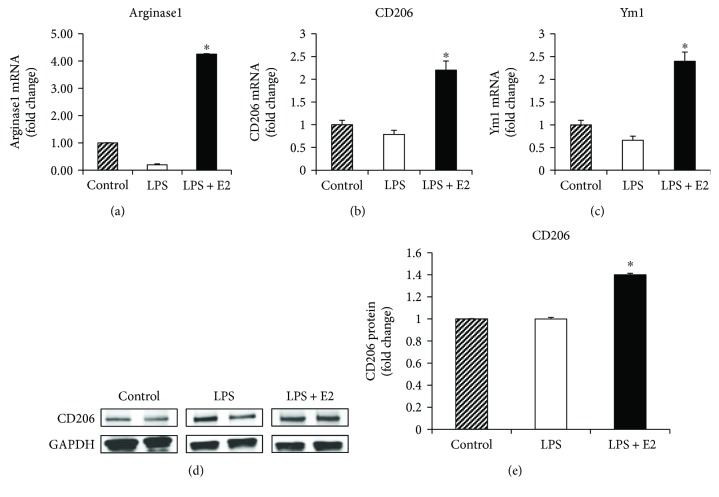
Upregulation of M2 phenotype markers by E2 in the LPS-activated BV2 microglia cells *in vitro*. (a–c) mRNA was collected from control, LPS-activated, and LPS activated + E2-treated BV2 cells at 16 hours after activation and treatment. Quantitative RT-PCR analysis of M2 markers, Arginase1, CD206, and Ym1, indicates a significant upregulation after LPS activation and E2 treatment. (d, e) Western blot analysis and quantification of the M2 marker, CD206, indicate a significant increase in expression after E2 treatment of activated BV2 cells (*n* = 5-6 per group) (^#^*p* < 0.05, control versus LPS, ^∗^*p* < 0.05, LPS versus LPS + E2).

To further confirm these findings, we examined whether E2 could directly modulate M1/M2 microglia polarization of BV2 microglia cells in culture by examining expression of M1 and M2 markers. As shown in Figures 6(a)–6(c), LPS treatment caused a robust increase in mRNA levels of all three M1 markers: CD86 (*p* < 0.001), iNOS (*p* < 0.001), and CD32 (*p* = 0.007), in BV2 microglia cells, and E2 treatment significantly attenuated this effect for CD86 (*p* < 0.001), iNOS (*p* = 0.001), and CD32 (*p* < 0.001). Western blot analysis of the M1 marker, iNOS (Figures 6(d) and 6(e)) (ANOVA: 6E *F* value = 17.303, *p* = 0.003), indicates that iNOS protein is significantly upregulated after LPS activation of BV2 microglia cells (*p* = 0.003), and this effect is significantly attenuated by E2 treatment (*p* = 0.011). Examination of gene expression for the M2 microglia markers, Arginase1, CD206, and Ym1, is shown in Figures 7(a)–7(c). As shown in Figures 7(a)–7(c), LPS treatment did not have a significant pattern of change for all three M2 markers, while E2 treatment significantly elevated the expression for Arginase1 (*p* < 0.001), CD206 (*p* = 0.002), and Ym1 (*p* = 0.000) from 2- to 4-fold versus controls. Western blot analysis of the M2 marker, CD206 (Figures 7(d) and 7(e)) (ANOVA: 7E *F* value = 12.323, *p* = 0.012), revealed that LPS had no significant effect upon protein levels of CD206 protein (*p* = 1.000), while E2 treatment caused a significant enhancement of CD206 protein levels in the LPS-activated BV2 microglia cells (*p* = 0.040).

**Figure 8:**
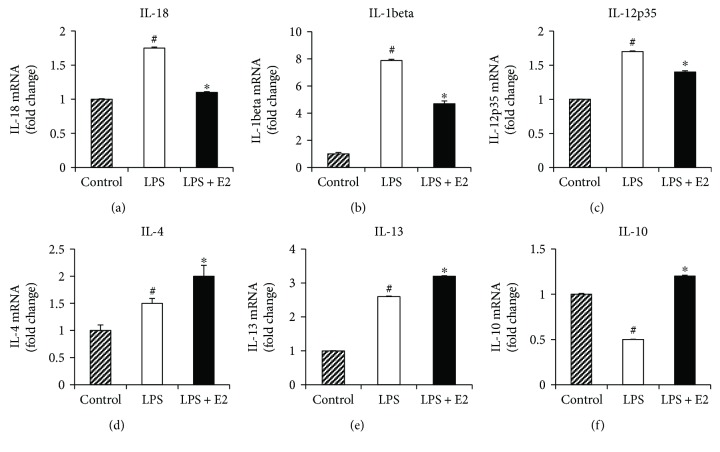
E2 regulates pro- and anti-inflammatory cytokine expression in BV2 microglia cells *in vitro.* mRNA was collected from control, LPS-activated, and LPS activated + E2-treated BV2 cells at 16 hours after activation and treatment. (a–c) RT-PCR analysis of proinflammatory cytokines, IL-18, IL-1beta, and IL-12p35, indicates that an LPS activation leads to a significant increase in their expression, and E2 treatment suppresses it. (d–f) RT-PCR analysis of anti-inflammatory cytokines, IL-4, IL-13, and IL-10, indicates that an LPS activation leads to a significant increase in their expression, and E2 treatment further enhances this expression of anti-inflammatory cytokines (*n* = 5-6 per group) (^#^*p* < 0.05, control versus LPS, ^∗^*p* < 0.05, LPS versus LPS + E2).

M1-polarized microglia are known to have enhanced expression of proinflammatory cytokines, while M2-polarized microglia have enhanced expression of anti-inflammatory cytokines and repair factors. Thus, we next examined the gene expression profile of both pro- and anti-inflammatory cytokines in LPS and E2-treated BV2 microglia cells. As shown in Figures 8(a)–8(c), LPS activation increased the gene expression of all three proinflammatory cytokines IL-18 (*p* = 0.001), IL-1*β* (*p* < 0.001), and IL-12p35 (*p* = 0.001) in BV2 microglia cells, while E2 treatment caused a significant attenuation of the LPS induction of the proinflammatory cytokines, IL-18 (*p* = 0.001), IL-1*β* (*p* < 0.001), and IL-12p35 (*p* = 0.001). Interestingly, LPS activation also increased mRNA levels of the anti-inflammatory cytokines, IL-4 and IL-13, but decreased expression of IL-10. In contrast, E2 treatment significantly increased mRNA levels for anti-inflammatory cytokines, IL-4 (*p* = 0.005), IL-13 (*p* = 0.001), and IL-10 (*p* = 0.001) as compared to LPS alone (Figures 8(d)–8(f)). Thus, our *in vitro* studies indicate that E2 could directly act on the microglia cells to regulate their activation and M1/M2 polarization via suppression of M1 phenotype markers and proinflammatory cytokines and elevation of M2 phenotype markers and anti-inflammatory cytokines.

### 3.4. E2 Attenuation of LPS-Induced IL-1*β* Is Correlated with a Switch to M2 Microglia Polarization In Vitro

**Figure 9:**
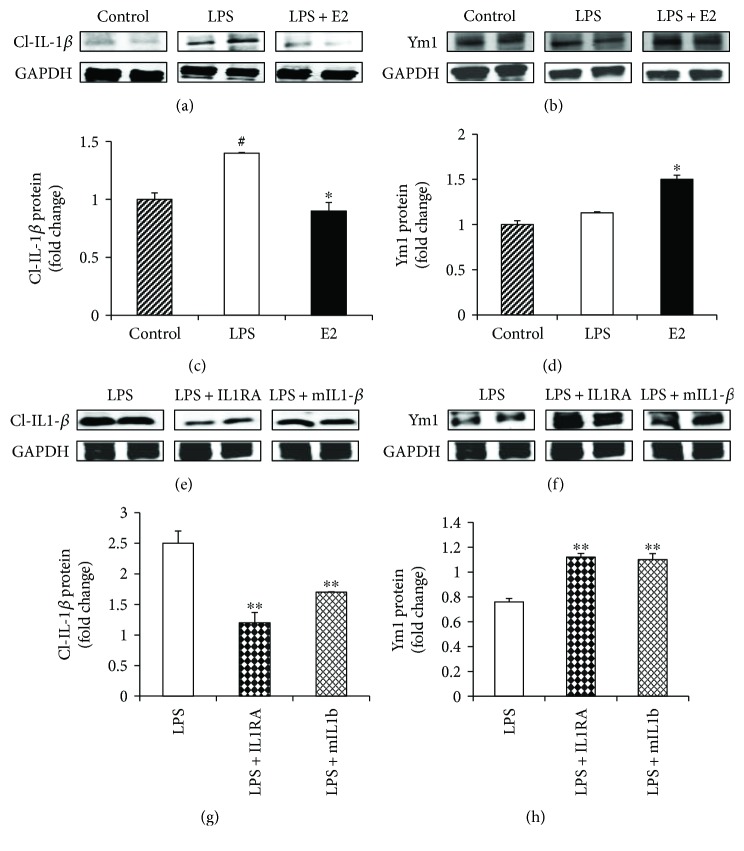
Effect of IL-1 receptor antagonist (IL1RA) and neutralizing monoclonal antibody for IL-1beta (mIL1*β*) on expression of M1 and M2 markers in LPS-activated BV2 microglia cells *in vitro*. (a, b) Western blot analysis of cleaved IL-1*β* and Ym1 indicates that E2 treatment suppresses M1 marker, cleaved IL-1*β*, and upregulates expression of M2 marker, Ym1. (c, d) Quantification of Western blot analysis indicates that LPS activation leads to a significant increase in M1 marker, cleaved IL-1*β* levels, and a significant suppression by E2. It further indicates that M2 marker, Ym1, is significantly upregulated by E2. (e, f) Western blot analysis of cleaved IL-1*β* and Ym1 indicates that IL1RA and neutralization of IL-1*β* suppress M1 marker, cleaved IL-1*β*, and upregulate expression of M2 marker, Ym1. (g, h) Quantification of Western blot analysis indicates that LPS activation leads to a significant increase in M1 marker, cleaved IL-1*β* levels, and a significant suppression by IL1RA and neutralization of IL-1*β*. It further indicates that M2 marker, Ym1, is significantly upregulated by IL1RA and neutralizing IL-1*β* treatments (*n* = 5-6 per group) (^#^*p* < 0.05, control versus LPS, ^∗^*p* < 0.05, LPS versus LPS + E2, ^∗∗^*p* < 0.05, LPS versus LPS + IL1RA or LPS + mIL1beta).

Previous work revealed that IL-1*β* could exert trophic effects upon neighboring microglia to induce M1 proinflammatory microglia activation [70]. Therefore, we hypothesized that E2 suppression of IL-1*β* could be one mechanism underlying its ability to induce a switch from M1 to M2 microglia phenotypes. To further explore this possibility, we examined E2 regulation of cleaved IL-1*β* at the protein level and determined whether downregulation of cleaved IL-1*β* by E2 was correlated with a switch to the alternative M2 microglia phenotype in BV2 microglia cells (as determined by examining protein levels of the M2 marker, Ym1). We also performed causation studies to determine whether blocking the IL-1*β* receptor with an antagonist (interleukin 1 receptor antagonist, IL1RA, 10 ng/mL for 16 hours) or immunoneutralization of IL-1*β* with a neutralizing antibody (10 ng/mL for 16 hours) would inhibit cleaved IL-1*β* levels and enhance M2 polarization of LPS-activated BV2 cells. As shown in Figures 9(a)–9(d) (ANOVA: 9C *F* value = 14.778, *p* = 0.005, 9D *F* value = 18.966, *p* = 0.003), Western blot analysis revealed that E2 suppressed the LPS-induced elevation of cleaved IL-1*β* (*p* = 0.008), an effect that correlated with E2 increasing protein levels of the M2 marker, Ym1 (*p* = 0.009). Furthermore, as shown in Figures 9(e)–9(h) (ANOVA: 9G *F* value = 31.382, *p* = 0.010, 9H *F* value = 28.938, *p* = 0.011), treatment with ILRA or immunoneutralization with a monoclonal antibody to IL-1*β* resulted in a significant attenuation of cleaved IL-1*β* (*p* = 0.013, *p* = 0.040) and a corresponding increase in the M2 marker Ym1 (*p* = 0.018, *p* = 0.026), indicating a switch to a M2 microglia phenotype.

### 3.5. E2 Attenuates Neurotoxicity of Activated BV2 Microglia Cells

**Figure 10:**
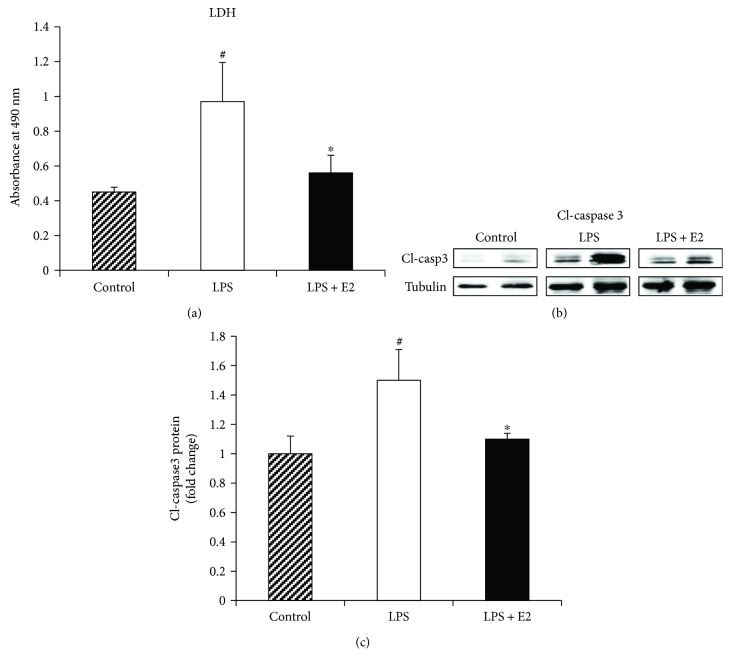
Neurotoxicity of LPS-activated BV2 microglia cells is inhibited by E2 *in vitro.* BV2 cells were activated using LPS. Conditioned media from control, LPS-activated, and LPS + E2-treated cells were transferred to hippocampal neuronal cell line, HT-22 cells. HT-22 cells were treated with these conditioned media, supernatant was harvested for LDH assay, and cells were harvested for cleaved-caspase3 analysis. (a) LDH levels using the LDH assay kit were determined in the control, LPS, and LPS + E2-treated groups. This indicates that LPS-activated cells have increased LDH release, and this is suppressed by E2 treatment. (b, c) Western blot analysis and quantification from HT-22 cells indicate that LPS group had increased cleaved-caspase3 levels which are significantly suppressed by E2 treatment (*n* = 5-6 per group) (^#^*p* < 0.05, control versus LPS, ^∗^*p* < 0.05, LPS versus LPS + E2).

Conditioned media from LPS-primed BV2 microglia cells are known to be neurotoxic to neuronal cells through activating inflammatory pathways [71]. To explore whether E2 treatment was able to attenuate the neurotoxicity of the LPS-primed BV2 cells, we utilized the HT-22 hippocampal neuronal cell line treated for 4 hr with conditioned media from control BV2 microglia cells, LPS-activated BV2 microglia cells, or LPS + E2-treated BV2 microglia cells. At the end of the treatment, the media was collected and tested for cytotoxicity using an LDH assay kit, or HT-22 cell lysates were collected to test for apoptosis using Western blot analysis for cleaved-caspase 3, a classical marker of apoptosis. As shown in Figure 10(a) (ANOVA: 10A *F* value = 7.507, *p* = 0.003), LDH assay results revealed that conditioned media from LPS-treated BV2 microglia cells were highly neurotoxic to HT-22 neuronal cells *in vitro*, as compared to conditioned media from control non-LPS-treated BV2 microglia cells (*p* = 0.000). Interestingly, the LDH assay results also revealed that conditioned media from LPS + E2-treated BV2 microglia cells had greatly reduced neurotoxicity on HT-22 cells, as compared to the LPS-treated BV2 microglia cell conditioned media (*p* = 0.001). It should be noted that we measured LDH in the conditioned medium of all three groups prior to adding the conditioned media to the HT-22 cells and found little to no LDH levels (data not shown). This further confirmed that the LDH release found in the media at the end of the incubation with the conditioned media was from HT-22 neurons. To further confirm our neurotoxicity results, we used a second marker that does not require measuring release of a factor into the conditioned media, but rather measurement of an “in cell” marker of apoptosis, cleaved-caspase 3. As shown in Figures 10(b) and 10(c) (ANOVA: 10C *F* value = 40.285, *p* = 0.000), Western blot analysis of HT-22 cell lysates revealed a significant increase in cleaved-caspase 3 protein levels in HT-22 cells that were exposed to the LPS-activated conditioned media (*p* = 0.001). In contrast, cleaved-caspase 3 expression was significantly reduced in the HT-22 cells that were subjected to LPS + E2-treated conditioned media (*p* = 0.001).

## 4. Discussion

To our knowledge, our study is the first to demonstrate that E2 can switch microglia polarization from a “proinflammatory” M1 state to a more “anti-inflammatory, repair” M2 state in the hippocampal CA1 region after GCI. Since we [12, 31, 72] and others [73–75] have reported previously that E2 exerts robust neuroprotection and improves cognitive outcome after GCI, the E2-induced switch in microglial polarization could contribute to the E2-induced neuroprotective effects and improved outcome after GCI. Indeed, in recent years, several studies have appeared showing that a switch in microglial polarization to the anti-inflammatory/repair M2 phenotype leads to improved outcomes in several neurodegenerative disorders [46, 76–78]. For instance, M2-polarized microglia exhibit better clearance of A*β* plaques in Alzheimer's disease [79], and enhanced M2 microglia polarization is correlated with decreased neurodegeneration in the substantia nigra in models of Parkinson's disease [79, 80]. Furthermore, administration of IL-4, a well-known anti-inflammatory cytokine and potent inducer of M2 microglia polarization, was shown to enhance M2 microglial polarization and improve functional and neurobehavioral outcomes following focal cerebral ischemia and intracerebral hemorrhage [81, 82]. Likewise, IL-4 treatment of brain slices *in vitro* enhanced M2 microglial polarization and was protective against oxygen glucose deprivation [83]. Additional studies using (+)-naltrexone to block Toll-like receptor 4 (TLR4) or a PPAR*ϒ* (peroxisome proliferator-activated receptor gamma) agonist, rosiglitazone, have also been reported to enhance M2 microglial polarization, leading to enhanced neuroprotection and improved cognitive outcome after cardiac arrest and intracerebral hemorrhage [84, 85]. Collectively, these studies, and our own findings suggest that a switch from M1 to M2 microglial polarization could help mediate the neuroprotective effects and improved outcome observed with E2 treatment in animals subjected to GCI.

It has been suggested previously that enhanced M2 polarization may be beneficial due, in large part, to a switch of production from M1 “proinflammatory cytokines” to M2 “anti-inflammatory cytokines and trophic factors,” thus decreasing inflammation and facilitating tissue and cellular repair [86, 87]. Indeed, using *in vitro* studies, we found that E2 switched microglial polarization of LPS-activated BV2 microglia cells from M1 to a predominantly M2 phenotype, with an associated switch from “proinflammatory” to an “anti-inflammatory” cytokine gene expression pattern in the activated BV2 microglia cells. This E2-induced switch in microglia polarization and cytokine expression appeared functionally important as it was correlated with a significant decrease in neurotoxicity of E2-treated LPS-activated BV2 microglial cells. Conceptually, reduced microglia neurotoxicity and enhanced anti-inflammatory cytokine production following E2 treatment could be an important mechanism contributing to E2-induced neuroprotection after GCI and in various neurodegenerative disorders. Interestingly, E2 has also been shown to enhance phagocytosis of hypoxia-activated BV2 microglia cells [61] and to enhance A*β* protein uptake in microglia derived from the human cerebral cortex [88]. These findings suggest that, in addition to decreasing neurotoxicity of activated microglia, E2 can also potentially enhance repair and clearance activities of microglia.

While microglial polarization has received considerable attention in recent years, the mechanisms underlying a switch from M1 to M2 microglia polarization remain poorly understood. Studies in focal cerebral ischemia animal models suggested a potential important role for anti-inflammatory cytokines like IL-10 and IL-4 in regulating microglia phenotype, as their administration induced a switch from M1 to M2 microglia polarization, as evidenced by increased production of the M2 marker, CD206, and decreased production of the M1 markers, TNF-*α* and IL-1*β* [78, 89, 90]. Likewise, in our current study, we provide evidence supporting a potential role for IL-1*β* as one of the potential supporting factors of M1 over M2 microglia polarization, as administration of IL1RA or a IL-1*β* neutralizing antibody led to upregulation of the M2 marker, Ym1, and corresponding downregulation of the proinflammatory cytokine, cleaved IL-1*β* in LPS-activated BV2 microglia cells. The functional importance of IL-1*β* in GCI pathology is evidenced by the fact that treatment with an IL-1*β* neutralizing antibody has been shown to enhance functional cognitive recovery after GCI [91]. Furthermore, in a previous study on the anti-inflammatory actions of E2, we also demonstrated that E2 can suppress NLRP3 inflammasome activation and its associated downstream IL-1*β* cytokine production in the hippocampus after GCI [23]. Based on this and our current findings, it is tempting to propose that E2 attenuation of NLPR3 inflammasome activation and its downstream product, IL-1*β*, may help facilitate the observed switch in microglial phenotype from M1 to predominately M2 after E2 treatment in GCI. Clearly, inflammation is a multimolecule cascade, and hence the possibility of involvement of multiple pathway regulation is likely and requires further study.

Additionally, although we did not explore the estrogen receptor type involved in the microglia regulatory effects of E2 in our study, we believe the E2 effects are likely to involve mediation by ER*β*, as BV2 microglia cells have been reported to only express ER*β*, and not ER*α* or GPER1 [61]. However, the situation *in vivo* may be more complex, as brain microglia under different conditions have been reported to express all three estrogen receptors, ER*α*, ER*β*, and GPER1 [92, 93]. Indeed, studies using ER*α* and ER*β* agonists in middle-aged, ovariectomized female rats have shown that activation of either ER*α* or ER*β* is capable of modulating the expression of neuroinflammatory genes in the frontal cortex, as well as modulating microglia-macrophage complement expression [94, 95]. Likewise, ER*α* knockout mice have spontaneous and enhanced microglia activation and an increase in proinflammatory cytokines, which correlated with an increased infarct size after focal cerebral ischemia [96, 97]. Furthermore, a potential role of GPER1 in E2 anti-inflammatory effects has also been suggested recently, as treatment of primary microglia with E2 or the GPER1 agonist, G1, was able to suppress LPS-induced proinflammatory cytokine production, and a GPER1 antagonist, G15, reversed the effects of E2 and G1 [60]. The suggestion that all three ERs may help mediate the anti-inflammatory effects of E2 *in vivo* is perhaps not surprising considering that all three ERs have been implicated in E2-induced neuroprotection [6, 25, 26, 50, 98]. Finally, it is intriguing and translationally relevant to note that current FDA-approved selective estrogen receptor modulators (SERMS) such as tamoxifen and raloxifene have been shown to reduce microglia activation, as well as proinflammatory cytokine and chemokine expression following neuronal injury [99]. Thus, SERMs or GPER agonists could be attractive therapeutic agents as they potentially could exert similar anti-inflammatory effects as E2, but with potential fewer associated negative side effects.

Finally, our study has some potential limitations that should be considered. First, for our in vitro studies, we chose to use the BV2 microglial cell line and LPS as the activator/inducer of microglial polarization and activation. These choices were made due to LPS being the most common microglial-inducing agent used in the literature and because BV2 cells provide ease of use and have been shown to mimic very closely primary microglia [43]. Nevertheless, confirmation of results in primary microglia would be advantageous, as would examination of other potential regulators such as oxygen glucose deprivation (OGD). Interestingly, previous work by another group has shown that E2 prevents the upregulation of proinflammatory cytokines after oxygen deprivation (hypoxia) in BV2 cells, suggesting that our findings using LPS may also be applicable to ischemia-relevant inducers such as oxygen deprivation/hypoxia [61]. An additional caveat is that we used young (3 months old) adult rats in our in vivo GCI studies. GCI is a hallmark of cardiac arrest, which can occur in young people, but is more frequent in aged individuals. At this point, we do not know if E2 would exert similar anti-inflammatory effects in aged animals as we observed in young animals after GCI. However, in previous studies, we did find that this same low-dose E2 replacement regimen was still neuroprotective against GCI in aged (9–12 months old) ovariectomized rats [32]. However, the anti-inflammatory and microglial phenotype regulatory effects of E2 in the aged animals were not studied in the previous study, and thus future studies will be needed to address this issue. Finally, we found that E2 had no significant effect upon microglial activation in the basal, noninduced state. This is consistent with our previous studies, where we also found no significant effect of E2 upon a variety of stress-induced and neurodegenerative factors in the basal nonischemic state (including Dkk1, JNK, pJNK, p53, Puma, and phospho-beta-catenin) [12, 72, 100]. Furthermore, others have examined E2 effects upon microglia *in vitro* in the basal state and confirmed that E2 does not induce either an M1 or M2 phenotype in the basal noninduced situation [101]. E2 also did not alter basal expression or activity of inflammatory markers of microglial activation, such as iNOS, NO, PGE2, and MMP-9 in microglial cells [102, 103], and did not alter LDH release or metabolic activity of microglial cells in the basal noninduced state [62]. Collectively, these studies suggest that E2 primarily regulates microglial polarization/activation in the induced (ischemic/injured) state.

In summary, the results of this study showed that M1 microglia polarization, as measured by M1 marker gene and protein expression, increased 3- to 8-fold in the hippocampus at days 3 and 7 following GCI, and this effect was strongly attenuated by E2 treatment. In contrast, M2 polarization showed little change in the hippocampus after GCI, but was robustly increased by E2 treatment. Mechanistic studies showed that E2 could act directly on BV2 microglia cells *in vitro* to (1) suppress M1 and increase M2 polarization after LPS treatment, (2) attenuate expression of proinflammatory cytokines while enhancing that of anti-inflammatory cytokines, and (3) reduce neurotoxicity of the BV2 microglia cells. Overall, findings from this study demonstrate that E2 can suppress M1 proinflammatory while enhancing M2 anti-inflammatory microglia polarization after GCI, which may contribute to the neuroprotective and anti-inflammatory effects of E2 after GCI.

## Figures and Tables

**Table 1:** Primers used for RT-PCR analysis of *in vivo* samples.

Gene	Forward primer	Reverse primer
TNF-*α*	5′CATCTTCTCAAAATTCGAGTGACAA 3′	5′GGGAGTAGACAAGGTACAACCC 3′
CD68	5′CCACAGGCAGCACAGTGGACA 3′	5′ TCCACAGCAGAAGCTTTGGCCC 3′
IL-1*β*	5′ CCCTGCAGCTGGAGAGTGTGG 3′	5′ TGTGCTCTGCTTGAGAGGTGCT 3′
Arginase1	5′ TCACCTGAGCTTTGATGTCG 3′	5′ TTCCCAAGAGTTGGGTTCAC 3′
CD206	5′ AGTTGGGTTCTCCTGTAGCCCAA 3′	5′ACTACTACCTGAGCCCACACCTGCT 3′
Ym1	5′ ACCCCTGCCTGTGTACTCACCT 3′	5′ CACTGAACGGGGCAGGTCCAAA 3′
CypA	5′ TATCTGCACTGCCAAGACTGAGTG 3′	5′ CTTCTTGCTGGTCTTGCCATTCC 3′

**Table 2:** Primers used for RT-PCR analysis of *in vitro* samples.

Gene	Forward primer	Reverse primer
CD86	5′ GACCGTTGTGTGTGTTCTGG 3′	5′ GATGAGCAGCATCACAAGGA 3′
iNOS	5′ CAAGCACCTTGGAAGAGGAG 3′	5′ AAGGCCAAACACAGCATACC 3′
CD32	5′ AATCCTGCCGTTCCTACTGATC 3′	5′ GTGTCACCGTGTCTTCCTTGAG 3′
Arginase1	5′ CAGAAGAATGGAAAGAGTCAG 3′	5′ CAGATATGCAGGGAGTCACC 3′
CD206	5′ CAAGGAAGGTTGGCATTTGT 3′	5′ CCTTTCAGTCCTTTGCAAGC 3′
Ym1	5′ CAGGGTAATGAGTGGGTTGG 3′	5′ CACGGCACCTCCTAAATTGT 3′
IL-18	5′ ACCAAGTTCTCTTCGTTGAC 3′	5′ TCACAGCCAGTCCTCTTAC 3′
IL-1*β*	5′ TACTGAACTTCGGGGTGATTGGTCC 3′	5′ CAGCCTTGTCCCTTGAAGAGAACC 3′
IL-12p35	5′ CTCCTAAACCACCTCAGTTTGGCCAGGGTC 3′	5′ TAGATGCTACCAAGGCACAGGGTCATCATC 3′
IL-4	5′ AGATGGATGTGCCAAACGTCCTCA 3′	5′ GGATTATG ACTGCCACTGCGAC 3′
IL-13	5′ TGAGGAGCTGAGCAACATCACACA 3′	5′ TGCGGTTACAGAGGCCATGCAATA 3′
IL-10	5′ CCAAGCCTTATCGGAAATGA 3′	5′ TTTTCACAGGGGAGAAATCG 3′
18S	5′ AACCTGCTGGTGTGTGACGTTC 3′	5′ CAGCACGAGGCTTTTTTGTTGT 3′

## Abbreviations

ASC: Apoptosis-associated speck-like protein
CARD: Caspase recruitment domain
CD32: Cluster of differentiation 32
CD68: Cluster of differentiation 68
CD86: Cluster of differentiation 86
CD206: Cluster of differentiation 206 or mannose receptor
DKK1: Dickkopf-related protein 1
E2: 17*β*-estradiol
ER-*α*: Estrogen receptor alpha
ER-*β*: Estrogen receptor beta
GCI: Global cerebral ischemia
GPER1: G-protein coupled estrogen receptor 1
Iba1: Ionized calcium-binding adapter molecule 1
IL-1*β*: Interleukin-1beta
IL1RA: Interleukin 1 receptor antagonist
IL-4: Interleukin-4
IL-12p35: Interleukin 12 p35 motif
IL-10: Interleukin 10
IL-13: Interleukin 13
IL-18: Interleukin 18
iNOS: Inducible nitric oxide synthase
JNK: c-Jun N-terminal kinase
LDH: Lactate dehydrogenase
LPS: Lipopolysaccharide
MMP-9: Matrix metallopeptidase 9
NLRP3: NOD-like receptor with a pyrin domain-3
NO: Nitric oxide
OGD: Oxygen glucose deprivation
p53: Tumor protein 53
PELP1: Proline-, glutamic acid-, and leucine-rich protein 1
pJNK: Phosphorylated c-Jun N-terminal kinase
PGE2: Prostaglandin E synthase
PPAR*γ*: Peroxisome proliferator-activated receptor gamma
puma: p53 upregulated modulator of apoptosis
P2X7: Purinergic receptor 7
TLR4: Toll-like receptor 4
TNF-*α*: Tumor necrosis factor alpha
TREM2: Triggering receptor expressed on myeloid cells 2
TXNIP: Thioredoxin interacting protein
Ym1: Chitinase 3-like 3.
